# Electrode-Geometry Control of Normal Electric-Field Distributions and Electrostatic Loading on Sessile-Droplet Interfaces: A Finite-Difference Study with a Finite-Element Cross-Check and Surrogate-Assisted Design Exploration

**DOI:** 10.3390/mi17080921

**Published:** 2026-07-30

**Authors:** Fahad Sulaiman Obaid, Muhammed Anaz Khan

**Affiliations:** Department of Mechanical Engineering, College of Engineering, University of Bisha, P.O. Box 551, Bisha 61922, Saudi Arabia; fobaid@ub.edu.sa

**Keywords:** normal electric field, electric capillary number, Maxwell stress, sessile droplet, off-axis pin electrode, three-dimensional electrostatics, finite-difference method, finite-element cross-check, Gaussian-process regression, Bayesian optimisation, grid convergence

## Abstract

Electrohydrodynamic emission from a sessile droplet depends on a coupled balance among electric traction, capillarity, gravity, charge transport and liquid motion. The present work addresses only the electrostatic-loading part of that problem. Verification-backed axisymmetric and three-dimensional Laplace solvers are used to map how parallel-plate, on-axis-pin, off-axis-pin and bipolar double-pin electrodes redistribute the normal electric field over a prescribed conducting water-droplet interface. The primary response is the dimensionless electric capillary number, Ca_E_ = ε_0_E_n_^2^R_v_/γ. To compare geometries on a common voltage scale, V_1_ is defined as the applied voltage at which the peak prescribed-interface loading reaches Ca_E_ = 1. V_1_ is a normalisation voltage and not a jetting or stability threshold. The axisymmetric solver reproduces the exact conducting-hemisphere solution to within 0.07% at the finest grid. The three-dimensional finite-difference results are mesh-assessed, and their normalised surface-field topology is cross-checked against an independently implemented Galerkin finite-element model. At 4 kV, the finite parallel-plate cell produces an apex enhancement of 3.24 relative to V/H. Replacing the plate with an on-axis 1 mm pin reduces the apex field by 39.5%, which corresponds to a 63% reduction in Ca_E_, and increases V_1_ from approximately 5.0 to 8.2 kV. Lateral pin displacement moves the surface-field maximum away from the apex and produces a broad nominal plateau near d = 5–7 mm, although the sub-grid steering distance remains sensitive to mesh and extraction settings. The bipolar double-pin configuration produces two symmetric surface-field maxima together with a near-null at the apex. This topology, but not its absolute magnitude, is reproduced by the finite-element cross-check. A Gaussian-process model interpolates the one-dimensional offset family accurately under leave-one-offset-out validation (R^2^ = 0.999). Four Bayesian-optimisation trials locate the broad steering plateau but show no visible evaluation-count advantage over random sampling in this one-dimensional test. A three-mesh study gives a reported field-magnitude mesh-sensitivity estimate of approximately 6.2% at the finest grid (rising to about 9.5% at the h = 0.20 mm production mesh) for the representative three-dimensional case, and an indicative combined-uncertainty band of approximately 10% is shown for V_1_ in the exploratory trade-off plot. Illustrative Young–Laplace profiles at contact angles of 70° to 110° preserve the comparative pin-versus-plate field reduction, whereas V_1_ varies by up to approximately 50%. A simplified Peek-law screening estimate places corona inception (the pin being cathodic) in the approximate range of 4.8–10 kV, which is comparable to the on-axis-pin V_1_, so gas discharge may intervene before large electrocapillary loading is reached in ambient air. The results establish electrode geometry as a controllable electrostatic-loading parameter while explicitly deferring coupled stability analysis and experimental validation. By resolving this loading on a single exact-solution-verified basis, the study quantifies electrode geometry as a control parameter that idealised enhancement factors and the nominal gap field cannot capture and provides a verified fixed-interface reference state for subsequent coupled electrohydrodynamic modelling.

## 1. Introduction

A conducting sessile droplet placed in a non-uniform electric field acquires induced free surface charge and experiences Maxwell traction. Under sufficiently strong coupled loading, the interface may deform, form a conical tip and emit a charged jet. That transition is governed not by electrostatics alone but by the interaction of electric traction with capillarity, gravity, charge transport, contact-line behaviour and liquid motion. Electrohydrodynamic emission underlies electrospray ionisation [1], high-resolution printing [2,3], aerosol and particle generation [4], microfluidic manipulation [5,6] and biosensing [7,8]. Reviews of electrohydrodynamic printing and spraying describe parallel-plate, pin–plate and related electrode arrangements [3,9,10]. Electrode geometry therefore determines the spatial distribution of the electric loading that a subsequent coupled stability calculation would use as a fixed-interface reference state.

Taylor’s classical analysis established the equilibrium cone angle of approximately 49.3° for an ideal electrified conical interface [11], and the Taylor–Melcher framework provides the standard leaky-dielectric closure for electrohydrodynamic interfaces [12]. Fully coupled phase-field, volume-of-fluid and cone-jet simulations demonstrate that deformation and emission depend on the evolving interface, flow and charge distribution [13,14,15]. The Rayleigh limit, q_R_ = (64π^2^ε_0_γR^3^)^1/2^, concerns instead the total-charge stability of an isolated conducting spherical drop [16,17]. The Taylor cone, the Rayleigh charge limit, local Maxwell-pressure scaling and the stability of a substrate-supported sessile drop are related electrocapillary concepts, but they are not interchangeable onset criteria. Accordingly, the present work reports electrostatic loading and does not assign a universal jetting threshold.

For a conducting hemisphere on a plane in an otherwise uniform field, the exact apex enhancement is E_apex_ = 3E_0_ [18]. Pin-plane fields are strongly non-uniform and depend on the finite electrode geometry [19]. Applied fields can deform sessile droplets substantially [20], and rapid induced-charge redistribution has been observed on water droplets [21]. These observations motivate direct mapping of the surface-normal field rather than reliance on the nominal gap field. The hypothesis examined here is that a pin can reduce the field delivered to the droplet apex by concentrating the global field near the pin tip, whereas lateral pin displacement can move the largest prescribed-interface loading away from the apex. Such field relocation may be relevant to candidate emission-site control in electrohydrodynamic printing [3,22], to local dosing and microfluidic analysis [5,7] and to cell-scale electric manipulation [23]. The actual emission location, however, can be established only by a coupled model or by experiment.

Sessile-droplet electrohydrodynamic loading must also be distinguished from confined-droplet splitting in microchannels. The former concerns an exposed, substrate-supported interface, whereas the latter concerns the breakup of a translating confined droplet. Both nevertheless illustrate the broader design principle that device geometry and applied forcing redistribute interfacial loading. Examples include valve-controlled splitting [24], dynamically reconfigurable pneumatic rails [25], voltage-controlled sorting and splitting [26] and the wider family of active and passive splitting mechanisms reviewed in [27].

To the authors’ knowledge, the main contribution of the present work is a common, axisymmetrically verified numerical framework that compares the prescribed-interface loading distribution (evaluated along the symmetry-plane trace for the non-axisymmetric cases) for a parallel plate, an on-axis pin, a one-parameter family of laterally displaced pins and a bipolar double-pin pair. The non-axisymmetric topologies are additionally checked using an independent finite-element implementation. The field maps are then linked to a deliberately limited surrogate and optimisation study. The novelty is therefore the geometry-to-loading-topology map together with its uncertainty-aware design interpretation. It is not a prediction of the coupled electrohydrodynamic jetting event.

To make this contribution explicit, the present framework differs from the two most common shortcuts, the idealised analytical enhancement factor and the nominal gap field E_0_ = V/H, in that it resolves the finite-electrode loading distribution (along the symmetry-plane trace for the non-axisymmetric cases) rather than a single idealised number; and it differs from fully coupled electrohydrodynamic simulation in that it isolates, verifies and makes reusable a fixed-interface electrostatic reference state that can be used to initialise, benchmark or interpret coupled models, at a cost of a few seconds per case. The resulting quantified advantage is developed in Section 3.9. On a common verified basis, an on-axis pin lowers the peak prescribed-interface loading by 63% in Ca_E_ and raises V_1_ from about 5.0 to 8.2 kV relative to the parallel plate, while off-axis and bipolar electrodes generate off-apex and two-maximum loading topologies that no idealised estimate reproduces.

### 1.1. Maxwell Stress and Perfect-Conductor Loading

The electrostatic Maxwell stress tensor is T_ij_ = ε(E_i_E_j_ − ½δ_ij_E^2^). With the unit normal directed from the liquid into the surrounding air, the normal traction jump for an air (out) to dielectric (in) interface is

ΔT_nn_ = ½ε_0_(E_n,out_^2^ − ε_r_E_n,in_^2^) + ½ε_0_(ε_r_ − 1)E_t_^2^.

For a perfectly conducting droplet, E_n,in_ = 0 and the tangential field at the surface vanishes, which gives σ_n_ = ½ε_0_E_n,out_^2^. The water droplet is approximated as an equipotential conductor. Using the representative values ε_r_ = 80 and σ = 5 × 10^−4^ S·m^−1^ gives τ_e_ = ε_0_ε_r_/σ ≈ 1.4 µs. This approximation is appropriate for a steady DC calculation, or for voltage changes much slower than τ_e_. It does not remove the need for charge-transport modelling during rapid transients [12,13,21].

### 1.2. Dimensionless Electrostatic Loading

The prescribed-interface loading is expressed by the electric capillary number,

Ca_E_(s) = ε_0_E_n_^2^(s)R_v_/γ, with R_v_ = (3V_d_/4π)^1/3^.

Here R_v_ is the volume-equivalent radius, which is identical for all equal-volume interface shapes. For V_d_ = 134 µL, R_v_ = 3.17 mm. The ratio of the local Maxwell stress to the capillary-pressure scale γ/R_v_ is ½Ca_E_. The quantity Ca_E_ is therefore a dimensionless loading measure and not a stability criterion.

For concise voltage comparison, V_1_ is defined as the applied voltage at which the maximum prescribed-interface loading reaches Ca_E_ = 1. Because E_n_ is linear in applied voltage for the fixed-geometry Laplace problem, Ca_E_ is quadratic in voltage, and V_1_ follows directly from a single field solution. The choice Ca_E_ = 1 is a normalisation convention only. It is not a demonstrated deformation, cone-formation or jetting boundary.

The Rayleigh total-charge limit is retained only as theoretical context. It applies to an isolated charged sphere and is not used to classify or threshold the present grounded sessile configuration [16,17].

### 1.3. Quantities Used to Compare Electrode Geometry

Two field quantities are reported: the apex normal field E_apex_ and the largest normal field E_n,max_ on the prescribed droplet interface (for the non-axisymmetric cases, the largest value along the symmetry-plane trace defined below). For the reference hemisphere, the plotted arc coordinate starts at one contact point and reaches the apex at s = πR/2. For non-axisymmetric cases, the surface trace is taken in the plane containing the droplet centre and the displaced pin. The nominal steering indicator is Δs = |s_peak_ − s_apex_|. Because Δs can be smaller than the grid spacing and because it depends on the surface-extraction procedure, it is interpreted qualitatively unless a mesh-converged interval is available.

### 1.4. Surrogate Models and Bayesian Optimisation

Geometry-conditioned surrogates have been applied to aerodynamic fields, shape optimisation, haemodynamic wall shear stress and dispersion calculations [28,29,30,31]. Four deliberately simple regressors are compared here: ordinary linear regression, a random forest, XGBoost and Gaussian-process regression with a Matérn-5/2 kernel. The exact voltage linearity is removed analytically, so that each surrogate models c(d) = E_n,max_/V. Validation withholds one complete offset at a time. Bayesian optimisation uses the Gaussian-process model with expected improvement [32] to explore the nominal steering indicator. Because the objective is one-dimensional, broad and numerically uncertain, the exercise is treated as a workflow demonstration rather than as evidence of algorithmic acceleration or of a unique physical optimum.

## 2. Materials and Methods

### 2.1. Geometry and Material Parameters

The lower electrode is a copper disc of 20 mm diameter and 2 mm thickness. A conducting water droplet of reference hemispherical radius R = 4 mm and volume 134 µL is in electrical contact with the disc. In the parallel-plate configuration, a grounded plate of 26 mm diameter and 1.5 mm thickness is positioned with its lower face at H = 10 mm above the lower disc. In the pin–plate configuration, a grounded pin of 1 mm diameter and 5 mm length has its tip at H = 10 mm above the disc, with a lateral offset in the range 0 ≤ d ≤ 9 mm. The bipolar double-pin configuration uses two identical pins at x = ±6 mm held at +20 and −20 kV, with the disc and droplet grounded.

The lower disc is modelled with its finite extent. On the finite-difference outer boundary, the potential satisfies an asymptotic Robin condition representing decay towards earth at infinity. The pin edge is assigned a nominal fillet radius r_tip_ = 0.10 mm in order to remove the mathematical sharp-edge idealisation. This radius is not fully resolved by the production three-dimensional grid, so pin-tip fields are not used as converged quantities. Water properties are ε_r_ = 80, γ = 7.28 × 10^−2^ N·m^−1^, ρ = 997 kg·m^−3^, and the assumed conductivity σ = 5 × 10^−4^ S·m^−1^ at 20 °C, consistent with the quoted surface tension γ = 7.28 × 10^−2^ N·m^−1^. The contact angles used in the Young–Laplace sensitivity study are illustrative values rather than measurements for a particular copper surface.

### 2.2. Axisymmetric Finite-Difference Solver

For the parallel-plate and on-axis pin–plate cells, φ(r,z) satisfies (1/r)∂_r_(r∂_r_φ) + ∂^2^_z_φ = 0. A flux-conserving five-point stencil retains the cylindrical 1/r term, and the r = 0 limit is imposed by mirror symmetry. Conductors carry Dirichlet conditions. Grid links cut by the analytic conductor boundary use a fractional-distance cut-link flux closure related to the classical embedded-boundary approach [33], with crossing fractions obtained by bisection. In the deposited implementation, each cut link is closed in flux form at its fractional crossing distance using the known surface potential, which is formally first-order accurate at the interface; the field accuracy is therefore established empirically by the exact-hemisphere verification and grid-convergence study of Section 2.4 rather than assumed from a nominal stencil order. The sparse linear system is solved either by direct factorisation or by algebraic-multigrid-preconditioned conjugate gradients.

The air-side normal field is evaluated along the exact surface normal. Bilinear interpolation is applied on the axisymmetric grid at distances d_ex_ and 2d_ex_ from the interface, followed by a second-order one-sided derivative that uses the known conductor potential. The baseline extraction distance is d_ex_ = 0.15 mm, and a sensitivity study over d_ex_ = 0.10–0.30 mm is reported in Table 1. Production axisymmetric field maps use h = 0.06 mm, as justified by the convergence study.

### 2.3. Three-Dimensional Solver and Finite-Element Cross-Check

Every off-axis single-pin case, together with the bipolar double-pin case, is solved on a uniform Cartesian grid using the same embedded-boundary construction. Trilinear interpolation is used for surface extraction, and a symmetry plane in y halves the domain. The linear system is solved by smoothed-aggregation algebraic-multigrid-preconditioned conjugate gradients to a relative residual below 10^−9^. The production spacing is h = 0.20 mm.

A separate continuous-Galerkin finite-element calculation with linear tetrahedra was implemented in scikit-fem for d = 6 mm and for the double-pin case. The finite-element model uses a large grounded outer box rather than the finite-difference Robin boundary, and it has a nominal tetrahedral spacing of approximately 0.5 mm. It is therefore used only to cross-check the normalised droplet-surface profile, together with the existence and approximate location of the field maxima. It does not independently validate the absolute field magnitude, the unresolved pin-tip field, or peak-position differences below 0.5 mm.

### 2.4. Verification, Convergence and Domain Sensitivity

**Exact-solution verification.** For a conducting hemisphere on a grounded plane in a uniform field E_0_, the exact potential is φ = −E_0_z(1 − R^3^/ϱ^3^), where ϱ = (r^2^ + z^2^)^1/2^, and the apex field is E_apex_ = 3E_0_. Applying the exact potential at the outer boundary yields a direct code-verification problem. The axisymmetric solver reproduces the apex factor with a finest-grid error of 0.07% and an observed second-order trend. A matching three-dimensional exact-hemisphere test in the Cartesian solver, with the same analytic potential imposed on the outer boundary, gives apex errors of 12.5%, 9.6% and 6.0% at h = 0.40, 0.30 and 0.20 mm and an observed order close to one, so the three-dimensional embedded-boundary scheme is first-order accurate; its production-mesh apex error of about 6% is consistent with the ≈10% combined uncertainty band used below.

**Axisymmetric grid convergence.** A three-grid sequence of h = 0.18, 0.12 and 0.08 mm with refinement ratio 1.5 gives reported Grid Convergence Index values of 0.21% for the parallel plate and 0.12% for the on-axis pin, following the standard procedure [34].

**Three-dimensional grid sensitivity.** For d = 6 mm, the sequence h = 0.30, 0.225 and 0.169 mm gives a field-magnitude mesh-sensitivity estimate of approximately 6.2% at the finest grid (rising to about 9.5% at the h = 0.20 mm production mesh); the apparent three-grid order is not in the asymptotic range, and the three-dimensional exact-hemisphere test above confirms that the Cartesian cut-link scheme is first-order accurate. Because the three-grid sequence also varied the domain extent, this value is treated as an indicative rather than a formally isolated grid-only estimate. The position-based steering indicator does not exhibit a clean asymptotic sequence because it is obtained from a sub-grid peak location. An indicative combined field-uncertainty band of approximately 10%, intended to cover grid, extraction and domain contributions and to exceed the ≈9.5% production-mesh estimate, is therefore shown for V_1_ in the exploratory trade-off plot. The double-pin calculation is assessed primarily through the persistence of the two-maxima topology and through the variation of the nominal maximum locations across meshes.

**Cross-solver and domain checks.** At d = 0, the Cartesian and axisymmetric apex fields differ by approximately 1% at h = 0.20 mm. For d = 6 mm, the nominal fitted finite-difference and finite-element peak positions differ by approximately 0.1 mm, but this agreement should be interpreted only within the coarser finite-element surface resolution of approximately 0.5 mm. Enlarging the axisymmetric domain from 25 to 40 mm changes the parallel-plate and pin–plate apex fields by less than 0.1%, and enlarging the d = 6 mm three-dimensional box laterally from 36 to 44 mm changes E_n,max_ by less than 0.5% (0.34%); the 28 mm box differs by about 2.1% from the 44 mm result and is not used as the converged reference.

### 2.5. Machine-Learning Pipeline

The ten single-pin offsets, d = 0–9 mm, are represented by c(d) = E_n,max_/V. All rows are taken from the same Cartesian-solver family for consistency, and the higher-resolution axisymmetric d = 0 solution is retained as a separate verification value. The regressors are ordinary least squares; a random forest with 400 trees, maximum depth 6 and random_state = 42; XGBoost with 400 estimators, maximum depth 3, learning rate 0.05, subsample 0.9 and random_state = 42; and Gaussian-process regression with a Matérn-5/2 kernel, scalar length-scale bounds of 0.3–20 mm, normalise_y = True, 15 optimiser restarts and random_state = 0. Leave-one-offset-out validation uses unrounded field coefficients, and aggregate metrics are reported in Section 3.5. All computations used Python 3.10 with NumPy, SciPy, scikit-fem, scikit-learn, XGBoost, PyAMG and Matplotlib; exact package versions are listed in the Zenodo README.

### 2.6. Bayesian Optimisation and Random Baseline

The nominal steering indicator Δs(d) is maximised. Each acquisition triggers a new three-dimensional solve rather than a lookup from the ten-point sweep. Four independent initial designs are restricted to d = 0.3–4.5 mm, so that the broad plateau near d = 5–7 mm is not included initially. Expected improvement uses ξ = 0.01 and is compared with random sampling at an equal solver budget. Because the objective is mesh-sensitive and because the study contains only four trials, the comparison is descriptive rather than a statistical performance test.

### 2.7. Exploratory Steering–Voltage Trade-Off

The two displayed responses are the primary-detector steering indicator Δs, for which larger values are preferable, and V_1_, for which smaller values are preferable. An indicative ≈10% numerical band is shown for V_1_. Steering uncertainty is not quantified uniformly over all offsets, so the plot is an exploratory trade-off visualisation rather than a formal interval-dominance or robust-Pareto analysis. A second peak detector is shown only at d = 3 mm, in order to expose detector sensitivity.

## 3. Results and Discussion

### 3.1. Electrostatic Field Maps

**Figure 1:**
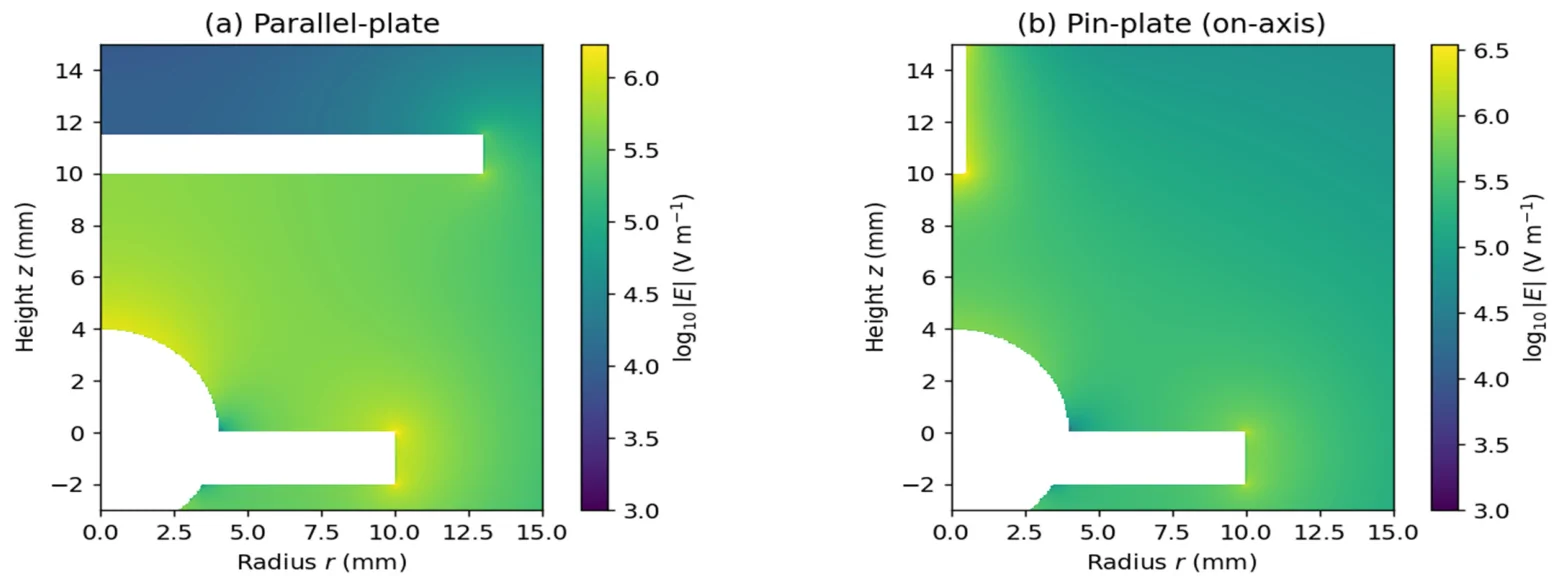
Electric-field-magnitude maps |E| on a logarithmic scale at V = 4 kV: (**a**) finite parallel-plate cell; (**b**) on-axis pin–plate cell.

The parallel-plate field is largest immediately above the droplet apex, whereas the global maximum in the pin–plate cell occurs near the grounded pin tip. On the droplet interface, however, the pin–plate normal field is lower than the parallel-plate apex field. These maps should not be interpreted as predictions of interface deformation because the droplet boundary is held fixed (Figure 1).

### 3.2. Voltage Scaling of Dimensionless Loading

**Figure 2:**
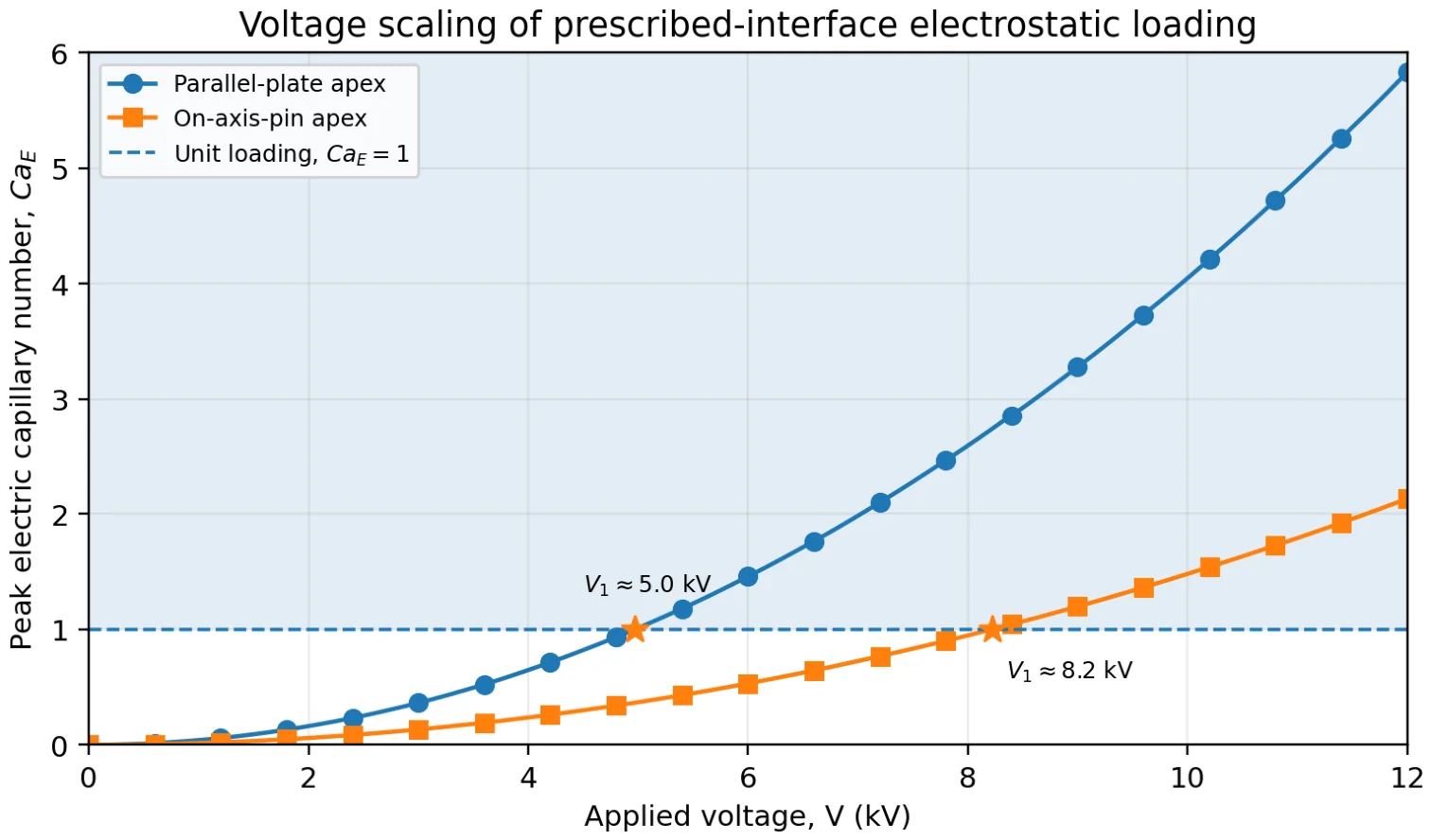
Peak prescribed-interface electric capillary number versus applied voltage. Stars mark V_1_, defined by max(Ca_E_) = 1. The shaded region is a comparison band and not a stability or jetting regime.

At 4 kV, the parallel-plate apex field is 1.296 × 10^6^ V·m^−1^, which corresponds to Ca_E_ ≈ 0.65. The finite-plate enhancement is 3.24 relative to E_0_ = V/H, close to but distinct from the ideal factor of three. The on-axis pin–plate apex field is 7.84 × 10^5^ V·m^−1^, which is 39.5% lower. Because Ca_E_ is proportional to E_n_^2^, the corresponding loading is approximately 63% lower. The normalisation voltages are V_1_ ≈ 5.0 kV for the parallel plate and 8.2 kV for the on-axis pin. These values compare voltage scaling only and are not onset predictions (Figure 2).

### 3.3. Effect of Lateral Pin Offset

**Figure 3:**
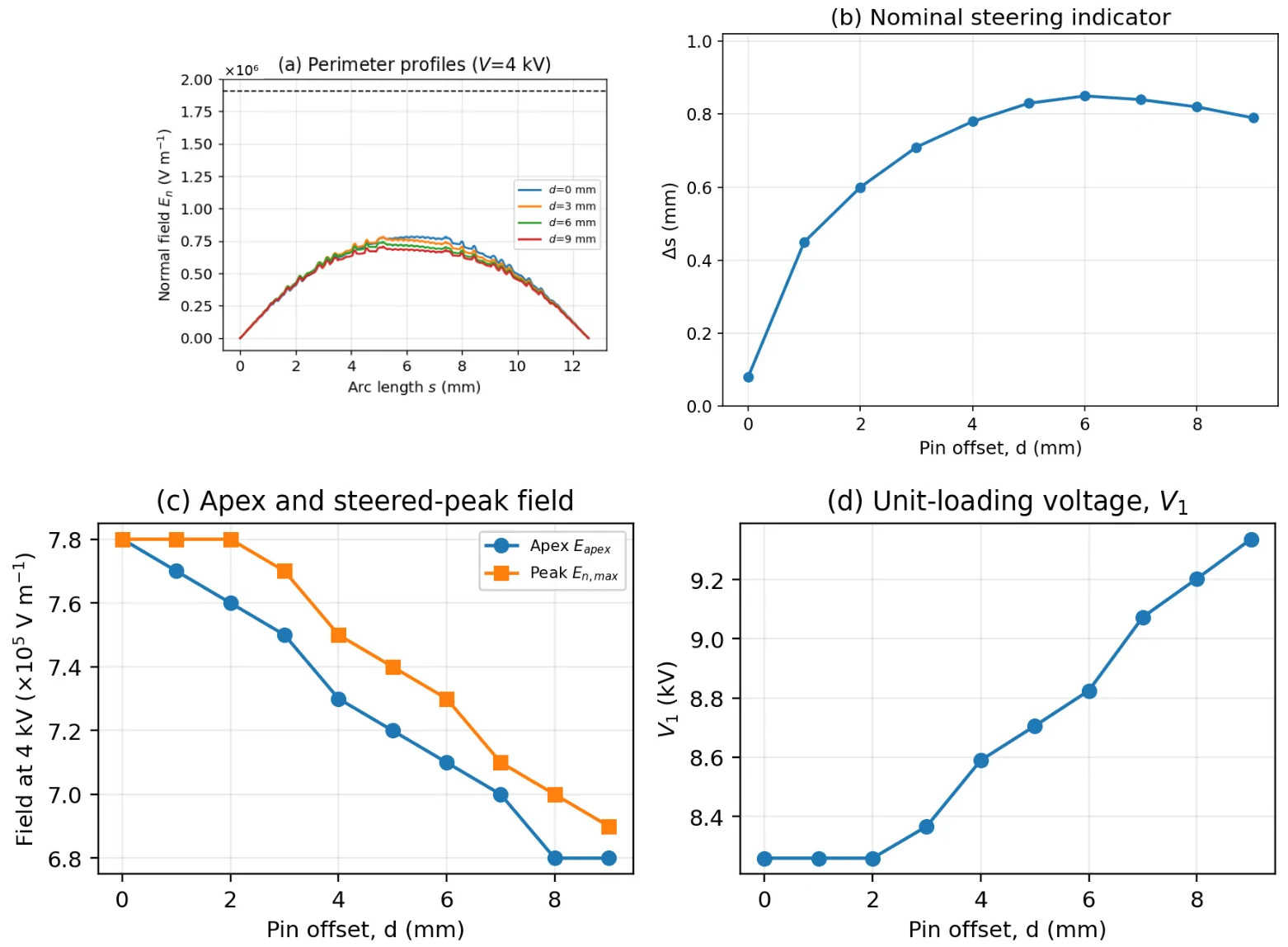
Single-pin offset study: (**a**) representative prescribed-interface profiles at 4 kV; (**b**) nominal steering indicator; (**c**) apex and largest surface-normal fields; (**d**) voltage V_1_ at which max(Ca_E_) = 1.

**Figure 4:**
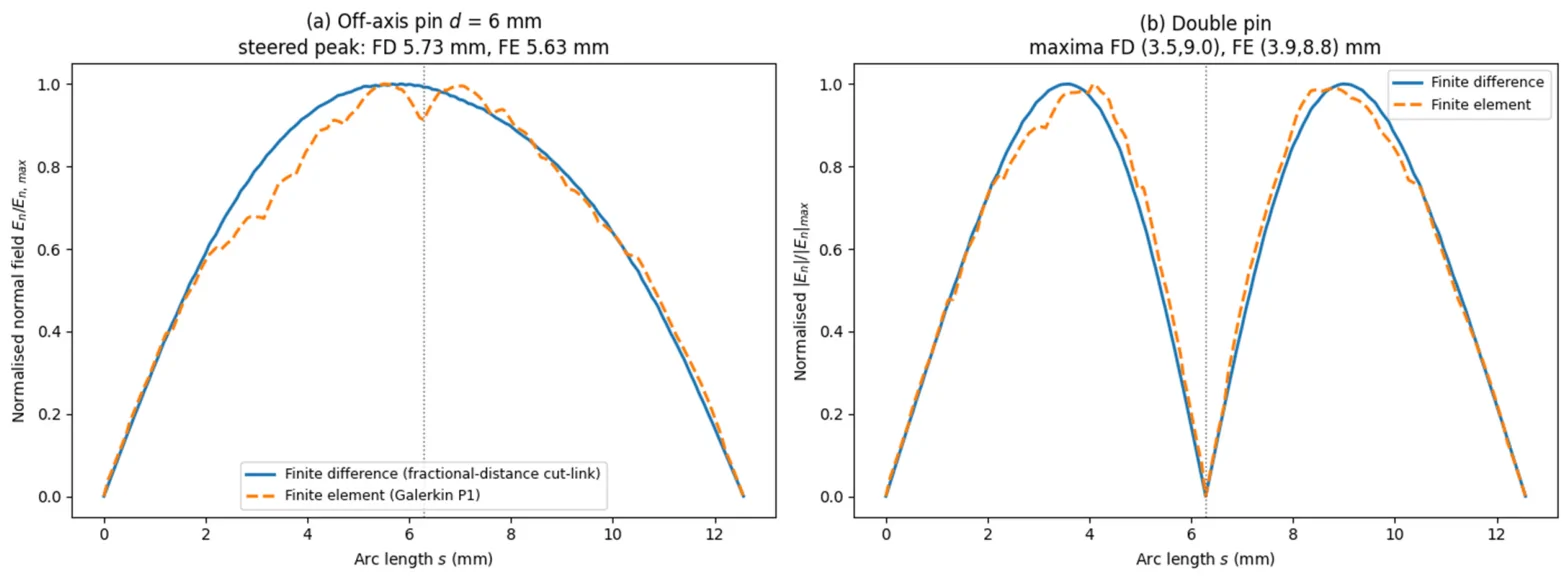
Normalised finite-difference and finite-element surface profiles: (**a**) off-axis pin at d = 6 mm; (**b**) bipolar double pin. The comparison tests topology and approximate peak position. The finite-element mesh is too coarse to validate absolute magnitude or sub-grid differences. The vertical dotted grey line marks the droplet-apex position.

The largest prescribed-interface field is nearly constant for d = 0–2 mm and then decreases as the pin moves away from the droplet centre. The primary detector gives Δs = 0.45, 0.60 and 0.71 mm at d = 1, 2 and 3 mm, respectively, together with a broad nominal plateau around d = 5–7 mm. A more robust peak detector gives approximately 1.0 mm at d = 3 mm. Because this detector dependence is comparable to the sub-millimetre signal, the exact steering distance is not converged. The defensible results are therefore the existence and direction of an off-apex maximum and the presence of a broad plateau, rather than a decimal-valued optimum. These single-pin trends are summarised in Figure 3 and Table 2, and the finite-difference surface-field topology is cross-checked against an independent finite-element solution in Figure 4.

For d = 6 mm, the two normalised profiles exhibit the same off-apex maximum. The fitted maxima differ nominally by 0.1 mm, but the meaningful agreement extends only to the finite-element surface resolution of approximately 0.5 mm. The same cross-check reproduces the presence of two maxima in the double-pin case.

### 3.4. Sensitivity to Gravity, Interface Shape and Contact Angle

For the reference water hemisphere of R = 4 mm, the Bond number is Bo = ρgR^2^/γ = 2.15 and the capillary length is l_c_ = (γ/ρg)^1/2^ = 2.73 mm. Gravity therefore cannot be neglected when the interface is interpreted as an equilibrium sessile shape. Equal-volume Young–Laplace profiles were computed for the illustrative apparent contact angles θ_c_ = 70°, 90° and 110°, and the numerical shape procedure was checked against the gravity-free spherical-cap limit. These contact angles constitute a sensitivity range rather than measured wettability data for the experimental copper surface (Table 3).

**Figure 5:**
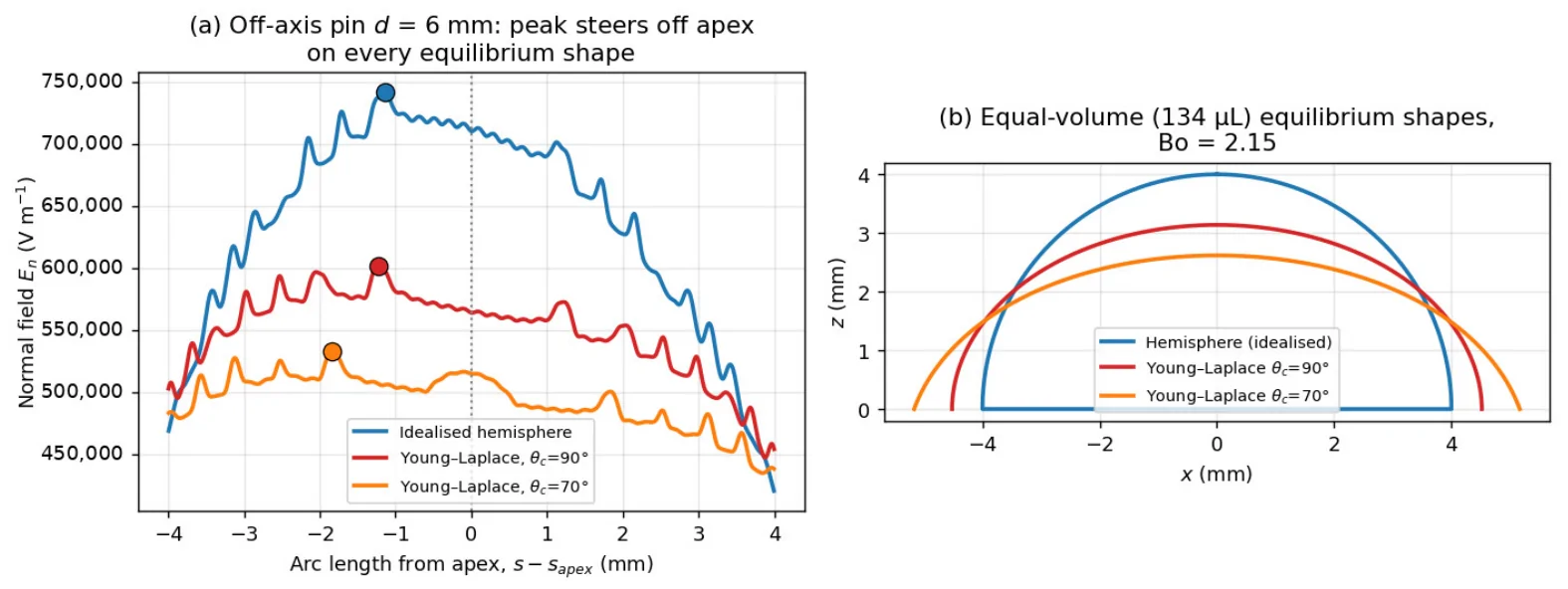
Illustrative shape calculations: (**a**) off-axis-pin prescribed-interface profiles for the reference hemisphere and for selected Young–Laplace profiles; (**b**) equal-volume interface shapes. Peak markers in panel (**a**) use the robust detector reported in Table 3.

The pin-to-plate apex-field reduction remains between 38.8% and 39.5%, so the comparative effect of electrode type is insensitive to the selected zero-field shape. The absolute fields are not insensitive: relative to the ideal hemisphere, the pin–plate V_1_ increases by approximately 17% to 50% across the illustrative profiles (Figure 5).

The off-apex single-pin maximum and the two-maxima double-pin topology persist on all evaluated profiles. Because the nominal displacement varies with mesh, extraction and geometry pathway, these calculations support qualitative topological robustness, but they do not establish that a flatter drop produces a quantitatively larger steering distance.

### 3.5. Surrogate Interpolation

The leave-one-offset-out results are based on unrounded c(d) values. Gaussian-process regression gives the highest interpolation accuracy within this single offset family. This result does not demonstrate transfer to another pin diameter, gap, droplet volume, contact angle or electrode topology (Table 4).

### 3.6. Bayesian Optimisation and Steering–Voltage Trade-Off

**Figure 6:**
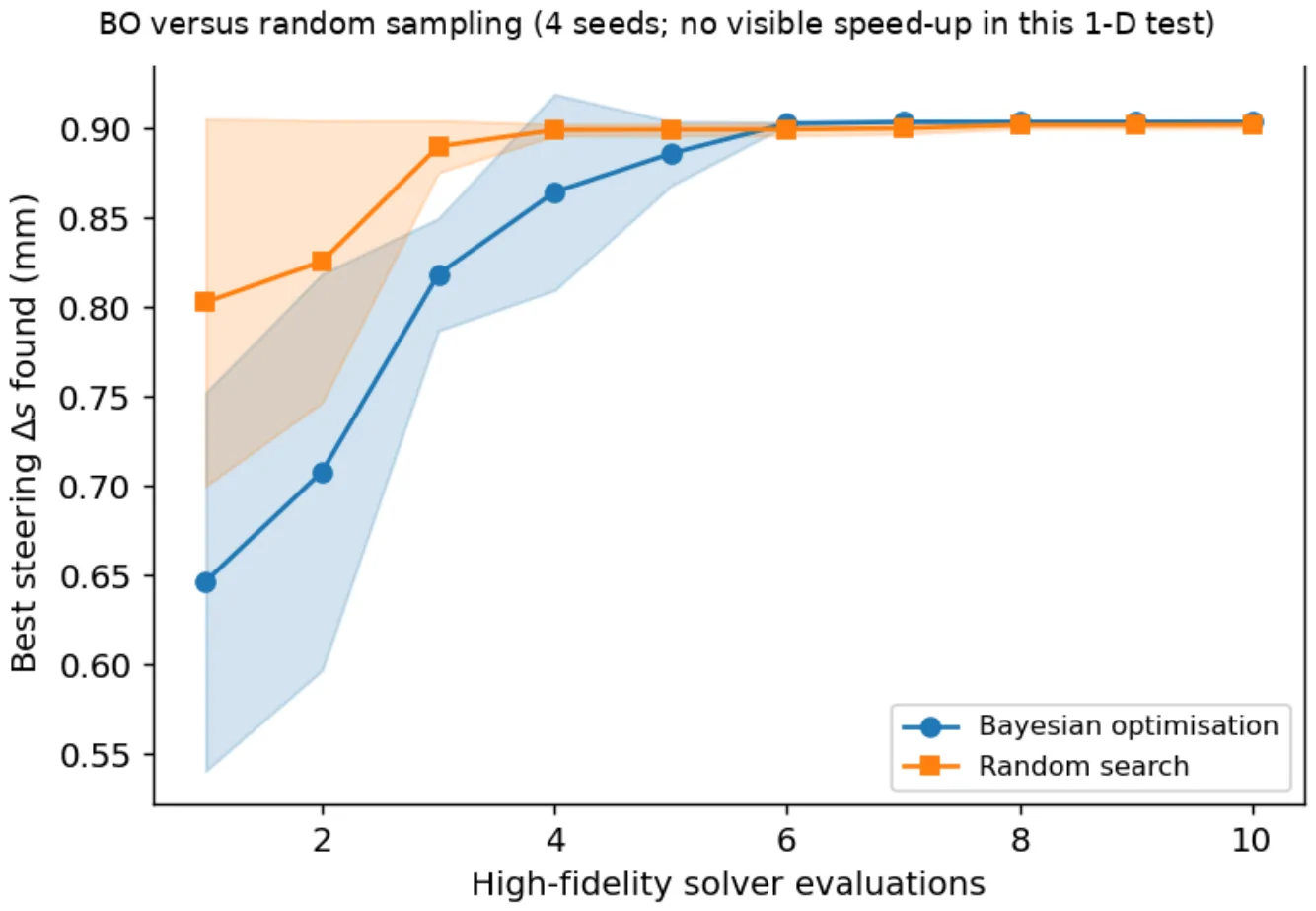
Bayesian optimisation and random sampling over four descriptive trials. The initial designs exclude the broad steering plateau.

Both strategies reach the broad nominal plateau within approximately five to six high-fidelity evaluations. No visible evaluation-count advantage is observed for Bayesian optimisation in this small one-dimensional problem. With only four trials and a mesh-sensitive objective, no statistical speed-up claim is made (Figure 6).

**Figure 7:**
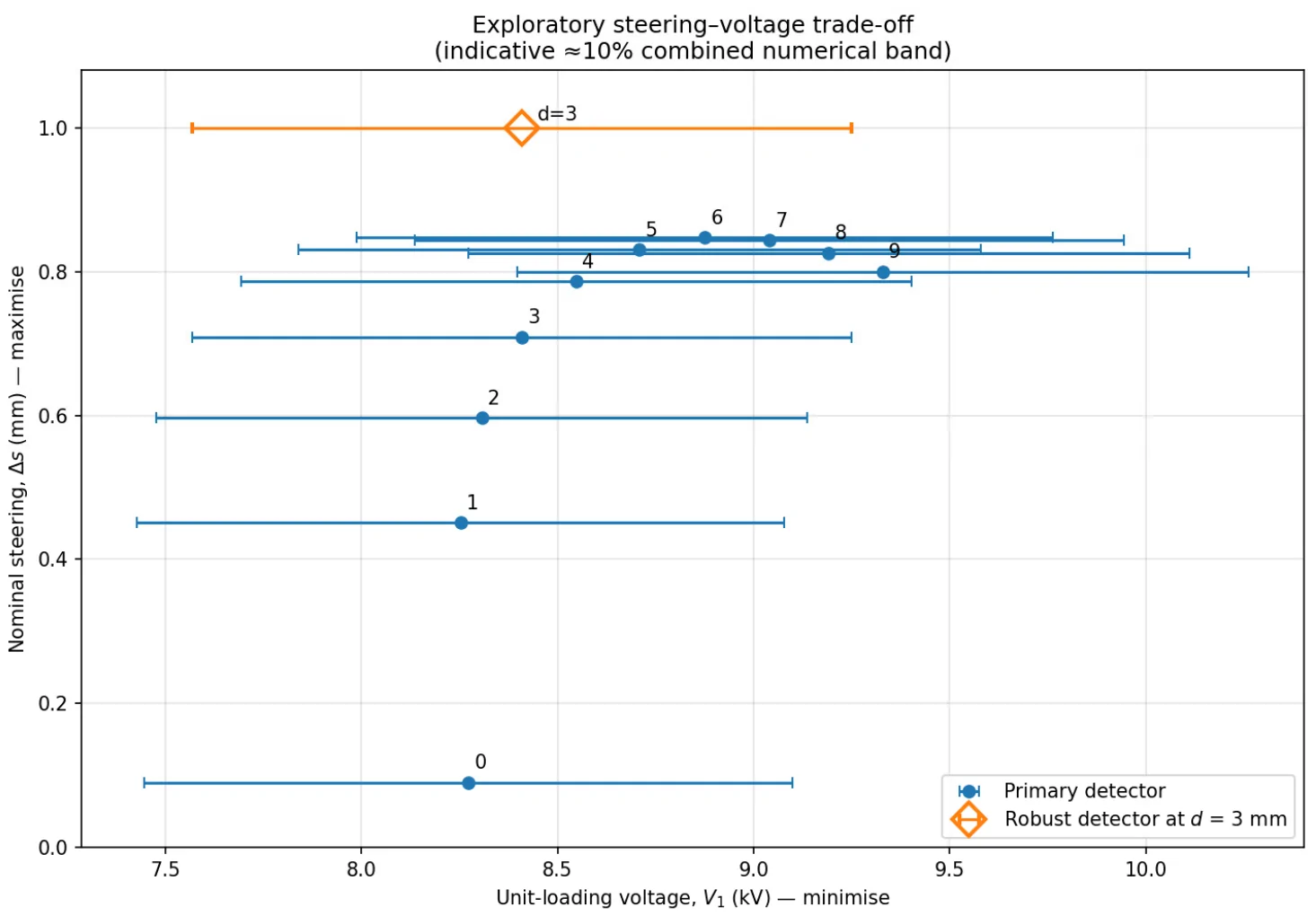
Exploratory steering–voltage trade-off. Filled markers use the primary detector, and horizontal bars show the indicative ≈ 10% numerical band for V_1_. The open marker shows the alternative robust-detector result at d = 3 mm. No formal steering-confidence interval or robust Pareto front is claimed.

The nominal data show that steering increases rapidly at small offsets while V_1_ changes only modestly and that this rise is followed by a broad plateau. Offsets of approximately 4–8 mm lie descriptively near the plateau, but detector sensitivity prevents a formal ranking based on Δs. The plot is therefore used only to illustrate the engineering trade-off, and no unique optimum, interval-dominance set or robust Pareto front is claimed (Figure 7).

### 3.7. Bipolar Double-Pin Field Topology

**Figure 8:**
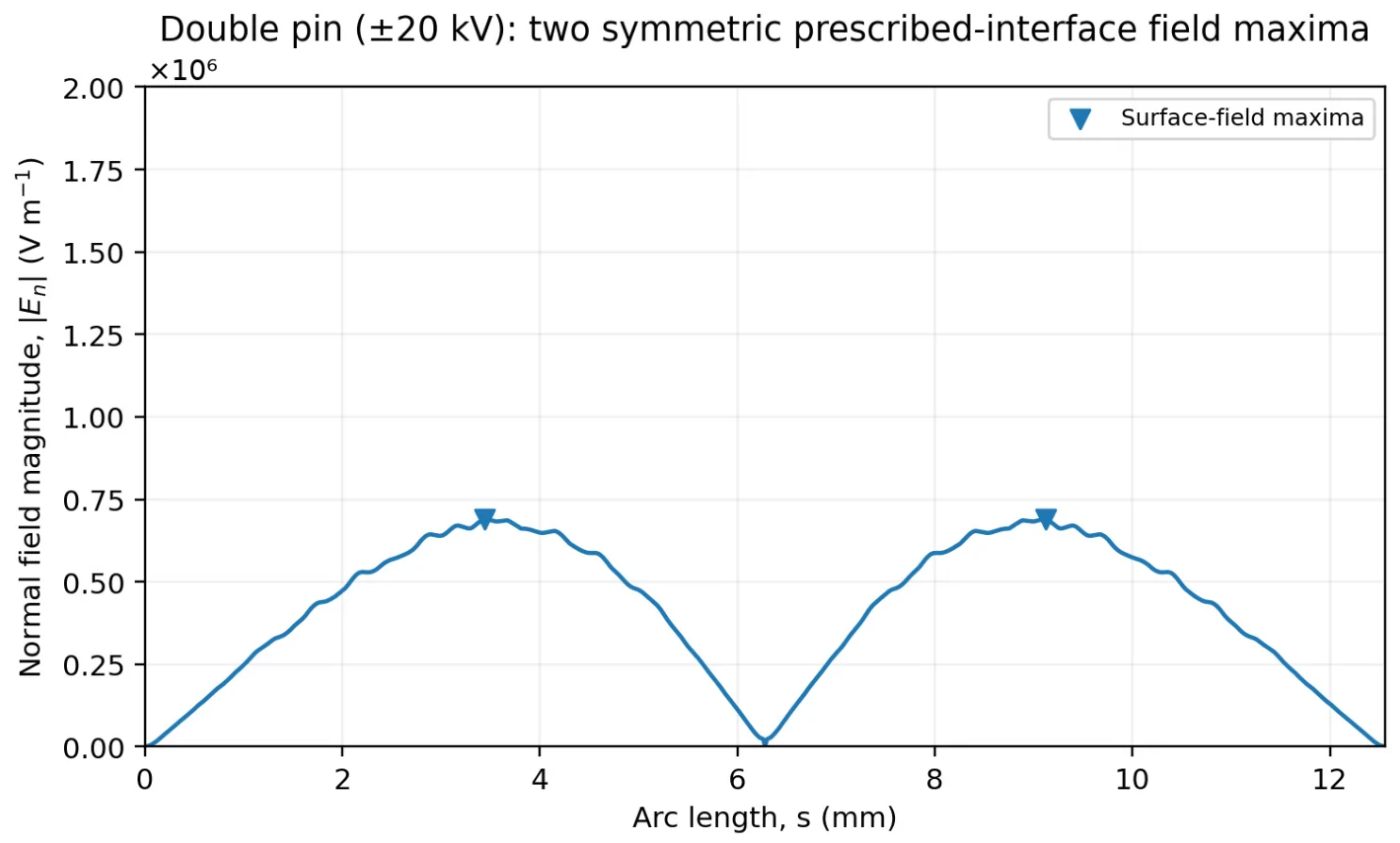
Bipolar double-pin configuration at ±20 kV: two symmetric maxima in the prescribed-interface normal field, together with a near-null at the apex.

The two maxima occur symmetrically about the apex, which is consistent with the antisymmetric electrode potentials. The peak field is approximately 6.9 × 10^5^ V·m^−1^, corresponding to Ca_E_ ≈ 0.18 at ±20 kV. Within the linear charge-free model, reaching Ca_E_ = 1 would require approximately 47 kV per pin. This extrapolation is not physically actionable in ambient air because corona, space charge and interface deformation would intervene. The calculation therefore establishes a two-maxima electrostatic topology and not simultaneous dual emission (Figure 8).

### 3.8. Corona and Gas-Discharge Screening

In the single-pin cell, the grounded pin faces the droplet-bearing disc, which is held at the positive applied potential, so the pin is cathodic; the following is therefore a polarity-neutral order-of-magnitude screening of the onset field magnitude rather than a positive-corona prediction. A simplified screening estimate is obtained from Peek’s empirical expression [35],

E_c_ = 30δ[1 + 0.301/(δr)^1/2^] kV·cm^−1^,

in which r is expressed in centimetres. For the nominal edge radius r = 0.10 mm = 0.01 cm and the air-density factor δ = 1, this gives E_c_ ≈ 120 kV·cm^−1^ = 1.2 × 10^7^ V·m^−1^. The computed pin-tip amplification is not mesh-converged and varies from approximately 1.2 × 10^3^ to 2.5 × 10^3^ V·m^−1^ per applied volt across the available refinements. Combining these values gives an inception range of roughly 4.8–10 kV. Peek’s law was developed for long cylindrical conductors, and the present finite rounded pin, together with polarity, surface condition, humidity and gap, introduces additional uncertainty. The estimate therefore indicates only that gas discharge may occur (as cathodic, i.e., negative, corona at the pin) at a voltage comparable to or below the on-axis-pin value of V_1_ ≈ 8.2 kV. It is not a validated inception prediction.

### 3.9. Novelty, Significance and Quantified Comparison with Common Approaches

The novelty and practical advantage of the present framework can be stated directly and quantitatively. Common treatments of the electrode–droplet problem fall into four groups: idealised analytical enhancement factors (for example, the conducting-hemisphere apex factor E_apex_ = 3E_0_), the nominal gap field E_0_ = V/H, fully coupled electrohydrodynamic simulation and direct experiment. The first two are closed-form conveniences that assume a single idealised geometry and therefore cannot rank electrode configurations, cannot locate the loaded region on the interface and cannot represent off-apex or multi-electrode loading. Coupled electrohydrodynamic simulations and experiments do capture emission, but they entangle the electrostatic loading with interface motion, charge transport and contact-line dynamics, are comparatively expensive and do not isolate the verified fixed-interface electrostatic reference state that can be used to initialise, benchmark or interpret coupled models. The present work addresses exactly this gap: it resolves the prescribed-interface normal-field distribution (along the symmetry-plane trace for the non-axisymmetric cases) for a parallel plate, an on-axis pin, a one-parameter family of off-axis pins and a bipolar double-pin pair on a single, exact-solution-verified numerical basis. On that common basis the finite parallel-plate apex enhancement is 3.24 rather than the idealised value of 3; replacing the plate with an on-axis 1 mm pin lowers the apex field by 39.5%, which is a 63% reduction in Ca_E_, and raises V_1_ from approximately 5.0 to 8.2 kV; lateral offset relocates the surface maximum away from the apex with a broad plateau near d = 5–7 mm; and the bipolar pair produces two symmetric maxima with an apex near-null. These geometry-to-loading relationships are quantitative, reproducible and, individually and collectively, unavailable from an idealised factor, from the gap field, or from any single coupled simulation. Table 5 summarises the difference from and the quantified advantage over each common approach. In this context, efficiency refers specifically to obtaining the fixed-interface electrostatic loading without the transient two-phase and charge-transport integration that a coupled VOF or phase-field solve carries. A direct wall-clock comparison with VOF is not reported because VOF does not produce a fixed-interface field as an output; the appropriate same-class benchmark, an independent finite-element solution on the identical interface, is given in Figure 4.

**Table 5:** Difference from and quantified advantage over common approaches to the electrode-geometry electrostatic-loading problem. All quantitative entries are taken from the present verified solutions.

Common Approach	What It Typically Provides	Limitation for the Electrode-Geometry Loading Problem	Present Work: Difference and Quantified Advantage
Idealised analytical factor (conducting hemisphere, E_apex_ = 3E_0_; Rayleigh charge limit)	One closed-form apex number for an idealised shape	Ignores finite electrode geometry; gives no off-apex or multi-electrode loading	Finite-electrode solution gives 3.24 rather than 3, the full axisymmetric profile and symmetry-plane traces for the non-axisymmetric configurations; the on-axis pin lowers apex loading by 39.5%, and off-axis and double-pin electrodes create new off-apex and two-maximum topologies
Nominal gap field, E_0_ = V/H	A single scalar field estimate	Cannot rank geometries or locate the loaded region on the interface	Verified E_n_(s) maps plus a dimensionless loading measure Ca_E_ and one comparison voltage V_1_; the on-axis pin lowers Ca_E_ by 63% and raises V_1_ from 5.0 to 8.2 kV
Fully coupled electrohydrodynamic simulation (VOF, phase-field, cone-jet)	Coupled interface deformation and jet emission	Entangles electrostatics with flow and charge transport; needs several closures; correspondingly more expensive per case	Provides a verified fixed-interface reference state for those models, in about 3–14 s per case on two CPU cores (Table 6)
Physical experiment	Real emission behaviour	Cannot isolate the electrostatic-loading term; costly; hard to sweep geometry	Cheap, openly documented geometry sweep (open Zenodo code and unrounded data); verified to 0.07% against the exact hemisphere in the axisymmetric solver; the three-dimensional scheme is first-order (≈6–9% production-mesh error) with a finite-element topology cross-check

**Table 6:** Illustrative recorded wall times on the authors’ Python 3.10 workstation using two CPU cores. The values are hardware- and implementation-dependent and are not platform-independent benchmarks.

Case	Spacing	Unknowns	Solver	Wall Time
Axisymmetric PP/on-axis PT	0.06 mm	≈3 × 10^5^	Sparse direct	≈3 s
3D off-axis pin, production	0.20 mm	≈9 × 10^5^	AMG-CG	≈5 s
3D off-axis pin, finest grid	0.169 mm	≈1.5 × 10^6^	AMG-CG	≈8 s
Independent FE topology check	≈0.5 mm tetrahedra	≈7 × 10^4^	Direct	≈14 s

The two most closely related electrode-geometry studies are those of Ahn et al. [36] and Khobaib et al. [37]. Ahn et al. compared pin–pin, planar–planar and pin–planar electrodes, experimentally and numerically, and found that pin electrodes increase the total acquired droplet charge by more than fourfold through local field intensification; that work, however, targets the total charge for droplet actuation in a microfluidic setting rather than the spatial distribution of the normal Maxwell-stress loading over a prescribed sessile interface, and it does not use a dimensionless electrocapillary measure or a common exact-solution-verified framework spanning an off-axis family and a bipolar topology. Khobaib et al. used a nonuniform electric field to probe the mechanical properties of particle-covered droplet interfaces, that is, electrode-generated field gradients as a rheological probe of an armoured interface, which differs from the present bare-conducting-interface loading map in both system and objective. The present contribution is therefore the geometry-resolved normal-field and electrocapillary loading topology, including off-apex relocation of the maximum and the two-maximum bipolar state on a common verified basis, rather than a total-charge comparison or an interfacial-mechanics measurement. Consistent with Ahn et al., the pin concentrates the field near its own tip; on the sessile interface studied here, that is precisely what lowers the normal loading delivered to the droplet apex.

The significance of these results follows from their role and their reusability. The prescribed-interface loading distribution provides a verified fixed-interface reference state that can initialise, benchmark or interpret coupled electrohydrodynamic calculations; in a fully coupled model, the field and traction evolve with interface shape, surface charge and charge transport, so this is a reference state rather than a universal boundary condition. The electrostatic loading is nonetheless usually either idealised away or left implicit inside an expensive coupled solve. Supplying it explicitly, verified and in dimensionless form across an electrode-geometry family therefore provides directly actionable design information for electrohydrodynamic and electrospray printing, droplet dosing and microfluidic manipulation, in which emission-site and loading control are central. The framework is also efficient and transparent: each verified case is solved in a few seconds on two CPU cores (Table 6), and the solver code, unrounded data, mesh studies and surrogate and optimisation pipelines are openly available, so the maps provide a verified fixed-interface reference state for initialising, benchmarking or interpreting coupled models. The accompanying Gaussian-process surrogate reproduces the offset family to R^2^ = 0.999 with a mean error of 0.07% under leave-one-offset-out validation, enabling near-instant interpolation of the geometry-dependent loading coefficient. The contribution is deliberately scoped to electrostatic loading rather than coupled jetting; within that scope it is complete, verified and quantitatively new.

### 3.10. Limitations and Outlook

- The interface is fixed. The calculation supplies electrostatic loading on a prescribed surface and cannot predict deformation, loss of stability, cone formation or jet initiation.
- The liquid is represented as a perfect conductor under steady DC conditions. Dielectric liquids and fast transients require leaky-dielectric charge transport together with tangential electric stress.
- The Young–Laplace profiles are zero-field illustrative shapes. They do not include electric deformation, measured copper wettability, contact-angle hysteresis or a pinned-contact-line experiment.
- The representative three-dimensional field magnitude has a reported mesh-sensitivity estimate of approximately 6.2% at the finest grid (rising to about 9.5% at the h = 0.20 mm production mesh), whereas an indicative ≈10% band is shown for V_1_ in the exploratory trade-off plot. The steering displacement is not mesh-converged.
- The independent finite-element model checks normalised topology only. Its coarse mesh and different far-field boundary do not validate absolute field magnitude or pin-tip fields.
- The Bayesian-optimisation and steering–voltage trade-off analyses use a one-dimensional, numerically uncertain objective. Their outputs are design-exploration demonstrations rather than transferable optima.
- The corona estimate is empirical and order-of-magnitude. Quantitative feasibility requires local mesh refinement, charge-transport modelling and experiment.

## 4. Conclusions

This study maps electrode-geometry effects on the prescribed-interface normal field and on the electric capillary number of a conducting sessile droplet. The defensible conclusions are as follows:

1. The finite parallel-plate cell gives E_apex_/E_0_ = 3.24 and V_1_ ≈ 5.0 kV. The on-axis 1 mm pin lowers E_apex_ by 39.5%, lowers Ca_E_ by approximately 63% at the same voltage and increases V_1_ to approximately 8.2 kV.
2. Lateral pin offset produces a reproducible off-apex maximum directed towards the pin. The nominal response has a broad plateau near d = 5–7 mm, but the sub-millimetre displacement is not sufficiently converged to define a unique geometric optimum.
3. The bipolar double-pin arrangement produces two symmetric prescribed-interface field maxima together with a near-null at the apex. This is an electrostatic topology result and not evidence of simultaneous emission.
4. Illustrative equal-volume Young–Laplace shapes preserve the comparative pin-versus-plate field reduction of 38.8% to 39.5%. Absolute loading and V_1_ remain sensitive to interface shape, and the selected contact angles are not measured substrate data.
5. Gaussian-process regression interpolates the ten-point one-dimensional offset family accurately under leave-one-offset-out validation. Four Bayesian-optimisation trials show no visible evaluation-count benefit over random sampling for this smooth one-dimensional plateau.
6. The three-dimensional field magnitude carries a reported mesh-sensitivity estimate of approximately 6.2% at the finest grid (about 9.5% at the h = 0.20 mm production mesh), and an indicative ≈10% band is shown for V_1_ in the exploratory trade-off plot. The finite-element calculation independently supports the normalised surface-field topology but not the absolute magnitude.
7. An order-of-magnitude Peek-law field screening for the cathodic pin gives a nominal range of 4.8–10 kV; this is not a polarity-specific corona-inception prediction. Gas ionisation may nonetheless occur at a voltage comparable to or below V_1_ in ambient air, which limits the direct physical interpretation of the charge-free high-voltage solutions.

Electrode geometry is thus a useful parameter for redistributing electrostatic loading. The present results should nevertheless be used as boundary-loading information for subsequent coupled electrohydrodynamic calculations and experimental validation, rather than as standalone jetting predictions. Because this prescribed-interface loading provides a verified fixed-interface reference state for initialising, benchmarking or interpreting coupled electrohydrodynamic calculations, resolving it on a common, exact-solution-verified basis, rather than through an idealised factor or the nominal gap field, is the central and quantitatively supported contribution of this work. Because the embedded-boundary solver is geometry-general and its cost scales with grid size rather than electrode complexity, application to more elaborate electrode arrays and to higher-dimensional design optimisation is a direct extension of the released framework. We identify this, together with coupling to a transient two-phase solver, as the natural next step.

## Figures and Tables

**Table 1:** Verification, grid-sensitivity and domain checks at 4 kV.

Test	Quantity	Result
Axisymmetric exact hemisphere	Apex factor	0.07% finest-grid error; *p* ≈ 2
3D Cartesian exact hemisphere	Apex factor	12.5%/9.6%/6.0% at h = 0.40/0.30/0.20 mm; *p* ≈ 1
Extraction distance, d_ex_ = 0.10–0.30 mm	Axisymmetric apex field	Variation < 1%
Axisymmetric GCI	PP/on-axis PT E_apex_	0.21%/0.12%
Cartesian versus axisymmetric, d = 0	E_apex_ at h = 0.20 mm	Difference ≈ 1%
3D grid sequence, d = 6 mm	E_n,max_	indicative mesh-sensitivity ≈ 6.2% (finest), ≈9.5% (prod.)
Exploratory field band	V_1_ in trade-off plot	≈10% indicative combined band
FD versus FE, d = 6 mm	Normalised peak location	Nominal difference 0.1 mm; interpret to ≈0.5 mm
FD versus FE, double pin	Number and approximate location of maxima	Same two-maxima topology; ≈0.5 mm resolution
Domain enlargement	Axisymmetric/3D field	<0.1%/<0.5%

**Table 2:** Three-dimensional single-pin sweep at V = 4 kV. Field magnitudes carry an indicative combined numerical band of approximately 10%. The Δs values are outputs of the primary peak detector and are not uncertainty intervals. Symmetry requires Δs = 0 at d = 0, where the reported 0.09 mm is detector residual. An alternative robust detector gives approximately 1.0 mm at d = 3 mm. The d = 0 Cartesian value V_1_ = 8.3 kV differs by approximately 1% from the independently computed axisymmetric value V_1_ = 8.2 kV.

d (mm)	E_apex_ (×10^5^ V·m^−1^)	E_n,max_ (×10^5^ V·m^−1^)	Primary-Detector Δs (mm)	V_1_ (kV)
0	7.8	7.8	0.09	8.3
1	7.7	7.8	0.45	8.3
2	7.6	7.8	0.60	8.3
3	7.5	7.7	0.71	8.4
4	7.3	7.5	0.79	8.6
5	7.2	7.4	0.83	8.7
6	7.1	7.3	0.85	8.8
7	7.0	7.1	0.84	9.1
8	6.8	7.0	0.83	9.2
9	6.8	6.9	0.80	9.3

**Table 3:** (**a**) Axisymmetric shape sensitivity for equal-volume 134 µL interfaces at V = 4 kV; (**b**) qualitative three-dimensional shape sensitivity at d = 6 mm. All Δs entries use the same robust peak detector, so that the comparison is like-for-like; for the reference hemisphere, the primary detector used in Table 2 gives 0.85 mm. The numerical values remain mesh- and extraction-dependent and are not treated as converged design predictions.

(a)					
Interface	Apex Height (mm)	PP E_apex_ (×10^6^)	PT E_apex_ (×10^5^)	Field Reduction (%)	PT V_1_ (kV)
Reference hemisphere	4.00	1.296	7.84	39.5	8.2
Young–Laplace, 70°	2.62	0.854	5.22	38.8	12.3
Young–Laplace, 90°	3.14	0.990	6.03	39.1	10.7
Young–Laplace, 110°	3.58	1.106	6.70	39.4	9.6
(**b**)					
**Interface**	**Apex Height (mm)**	**Robust-Detector Δs (mm)**	**Single-Pin Topology**	**Double-Pin Topology**	
Reference hemisphere	4.00	≈1.1	Off-apex maximum persists	Two maxima; nominally ±2.9 mm	
Hemisphere through profile pathway	4.00	≈1.1	Pathway-control result	Not evaluated	
Young–Laplace, 70°	2.62	≈1.8	Off-apex maximum persists	Two maxima; nominally ±3.6 mm	
Young–Laplace, 90°	3.14	≈1.2	Off-apex maximum persists	Two maxima; nominally ±3.0 mm	
Young–Laplace, 110°	3.58	≈1.3	Off-apex maximum persists	Not evaluated	

**Table 4:** Aggregate leave-one-offset-out metrics for c(d) = E_n,max_/V. Errors are computed from unrounded values; the two error columns are absolute errors normalised by the sample mean of c(d), not pointwise relative errors.

Model	R^2^	Mean Absolute Error (% of Mean)	Maximum Absolute Error (% of Mean)
Linear regression	0.941	0.76	2.62
Random forest, 400 trees	0.943	0.85	2.19
XGBoost	0.72	1.9	4.8
GPR, Matérn-5/2	0.999	0.07	0.40

## Data Availability

The numerical results in this manuscript depend on custom finite-difference, finite-element, Young–Laplace, machine-learning and plotting scripts. The solver code, the unrounded data underlying the reported tables and the mesh sequences are available in the Zenodo repository at https://doi.org/10.5281/zenodo.21547970. The deposit contains the axisymmetric and three-dimensional finite-difference Laplace solvers, the independent Galerkin finite-element cross-check, the augmented Young–Laplace shape solver, the surrogate and Bayesian-optimisation pipelines with fixed random seeds, the computed data, README documenting software versions, solver tolerances and the two peak detectors used for the steering indicator. This archived version (v1.0.2) also provides the figure-generation scripts and the corrected corona and threshold metadata.

## Nomenclature

Latin symbols		
Symbol	Description	Units
a	Pin radius (half of the 1 mm pin diameter)	m
Bo	Bond number, ρgR^2^/γ	–
c(d)	Geometry coefficient, E_n,max_/V	m^−1^
Ca_E_	Electric capillary number, ε_0_E_n_^2^R_v_/γ	–
d	Lateral offset of the pin axis from the droplet centre	m
d_ex_	Surface-normal field-extraction distance	m
E_0_	Nominal gap field, V/H	V·m^−1^
E_n_, E_t_	Normal and tangential field components	V·m^−1^
E_apex_	Normal field at the droplet apex	V·m^−1^
E_n,max_	Maximum normal field on the prescribed interface	V·m^−1^
H	Pin-tip or upper-plate height above the bottom electrode	m
h	Uniform grid spacing	m
l_c_	Capillary length, (γ/ρg)^1/2^	m
N	Number of discrete unknowns	–
q_R_	Rayleigh total-charge limit for an isolated spherical drop	C
R	Radius of the reference hemispherical interface	m
R_v_	Volume-equivalent radius, (3V_d_/4π)^1/3^	m
R^2^	Coefficient of determination	–
r, z	Cylindrical coordinates	m
s	Surface arc-length coordinate in the electrode-offset plane	m
s_peak_	Arc-length position of the maximum normal field	m
V	Applied direct-current voltage	V
V_1_	Voltage at which max(Ca_E_) = 1; comparison only	V
V_d_	Droplet volume	m^3^
x, y, z	Cartesian coordinates	m
Greek symbols		
Symbol	Description	Units
γ	Surface tension	N·m^−1^
Δs	Nominal steering displacement from the apex	m
δ_ij_	Kronecker delta in the Maxwell stress tensor	–
δ	Air-density correction factor in Peek’s empirical corona relation	–
ε_0_	Vacuum permittivity	F·m^−1^
ε_r_	Relative permittivity of the liquid	–
ϱ	Spherical radial distance, (r^2^ + z^2^)^1/2^, used in verification	m
θ_c_	Illustrative equilibrium contact angle	deg
μ, s^2^	Gaussian-process posterior mean and variance of c(d)	m^−1^, m^−2^
ξ	Expected-improvement exploration parameter	–
ρ	Liquid density	kg·m^−3^
σ	Electrical conductivity of the liquid	S·m^−1^
σ_n_	Normal Maxwell stress, ½ε_0_E_n_^2^	Pa
τ_e_	Charge-relaxation time, ε_0_ε_r_/σ	s
φ	Electrostatic potential	V

## Abbreviations

BO, Bayesian optimisation; CG, conjugate gradients; DC, direct current; DP, double pin; EHD, electrohydrodynamic; EI, expected improvement; FD, finite difference; FE(M), finite element (method); GCI, Grid Convergence Index; GPR, Gaussian-process regression; ML, machine learning; PP, parallel plate; PT, pin–plate; RF, random forest; VOF, volume of fluid.
